# Analysis of split tooth as an unstudied reason for tooth extraction

**DOI:** 10.1186/1756-0500-7-630

**Published:** 2014-09-10

**Authors:** Ifueko Patience Osaghae, Clement Chinedu Azodo

**Affiliations:** Department of Oral and Maxillofacial Surgery, Central Hospital, Benin City, Nigeria; Department of Periodontics, School of Dentistry, University of Benin, P.M.B. 1154 Ugbowo, Benin City, Edo State Nigeria

**Keywords:** Trauma, Split tooth, Tooth extraction, Dental clinic

## Abstract

**Background:**

Split tooth is an unstudied reason for tooth extraction. The purpose of this study was to determine and analyze split tooth as a reason for extraction in a dental clinic in Benin City.

**Methods:**

The prospective study was carried out on 669 patients having tooth extraction between May, 2005 and December, 2012. Over the period of the study, diagnosis and tooth extraction were done by three dentists of more five years practice experience. The indications for tooth extraction were noted with specific interest on those diagnosed as split tooth without restoration. Data was entered into Microsoft excel, sorted and transported into SPSS (SPSS version 16.0, Chicago, IL, USA).

**Results:**

Split teeth constituted for 39 (5%) of extracted teeth. This 39 extractions were done in 38 patients meaning that two split teeth were extracted on separate occasions from same patient. The majority 23 (61%) of extracted split tooth were done in patients in the fifth decade of life. More of the split tooth extraction were performed in males 28 (72%) than females 11 (28%). Overall mandibular teeth were more affected than the maxillary teeth and the most affected teeth were mandibular second molars 23 (59%) while the least affected were the mandibular first premolars 1 (3%) and the third molars 1 (3%). The reported masticatory accident as aetiology, were biting on stone 21 (53%) or piece of bone 10 (26%) while eating. A few 3 (8%) were suspected bruxists. The majority 25 (65%) visited the dental clinic 3–6 months after the incident and onset of symptoms.

**Conclusion:**

Split tooth constitute a reasonable common reason for tooth extraction and this was most common in the fifth decade of life. It is therefore important to improve early diagnosis of a cracked tooth in order to prevent the progression of the crack tooth to split tooth.

## Background

The retention of a complete dentition throughout life is one of the main goals of the dental profession [1] and the major objective of dental care is the prolongation of the life span of the dentition by either preventive or conservative treatment [2]. However tooth extraction has remained the most common oral procedure in oral healthcare setting in developing countries [3].

Trauma has been reported as the third most common reason for tooth extraction after dental caries and periodontal disease [3]. This trauma may either be in form of unrestorable tooth fracture or tooth in jaw fracture line. The unrestorable tooth fractures may occur slowly over time or as impact trauma or slowly over time. When a fracture extends through both marginal ridges usually in a mesiodistal direction splitting the tooth completely into two separate segments, it is considered a split tooth [4].

The term split tooth is defined as a complete fracture initiated from the crown and extending subgingivally, usually directed mesiodistally through both of the marginal ridges and the proximal surfaces. Split tooth is the evolution of a cracked tooth, though a split can occur suddenly, it results more commonly from long-term growth of an incomplete cracked tooth [5]. It is therefore considered as one of the five specific variations of cracked teeth alongside craze line, fractured cusp, cracked tooth and vertical root fracture according to the classification by American Association of Endodontists (AAE) [4]. Split tooth has been reported to constitute 10.0% of longitudinal fractured unrestored teeth according to the well-defined criteria of the AAE [6]. Since split tooth results from cracked tooth, early detection of a cracked tooth will facilitate the provision of the correct treatment and patient management which will prevent its propagation to a split tooth [7, 8]. However, the crack tooth is difficult to diagnose as the crack are usually invisible to the naked eye and undiscloseable with staining in the early stages [9].

Split tooth occurs from masticatory accidents, such as sudden and unusually high biting force on a hard, rigid object like piece of bone, incomplete fusion of areas of calcification, excessive removal of tooth structure during cavity preparation and parafunctional habits such as bruxism [10]. Split tooth is usually characterized by acute pain on mastication and at the time the patient presents in the clinic with a split tooth, diagnosis of the condition is obvious to patient and dentist; a split tooth will show mobility with wedging forces and the mobile segment will extend well below the cementoenamel junction. Split tooth can never be saved intact, but the prognosis and treatment depends on the position and the apical extent of the crack. If the crack is deep apically, the tooth must be extracted but if it extends only to the middle or cervical third of the root, the smaller mobile segment can be removed and the remainder of the tooth, restored. The review of literature neither reveal any study on split teeth in Nigeria and West Africa nor any epidemiological data in other parts of the world.

The purpose of the study was to determine and analyze split tooth as a reason for tooth extraction.

## Methods

The protocol for this study was reviewed and approved by the Ministry of Health, Benin-City, Edo State, Nigeria which was in compliance with the Helsinki Declaration. Written informed consent was obtained from the adult participants (those aged ≥18 years) and parents or guardian of underage participants (those less than 18 years). The prospective study was conducted on the extracted teeth in a dental clinic in Benin City between May, 2005 and December 2012. Benin City is the capital of Edo State, an ancient with population of approximately 1.1 million inhabitants. The age, gender and indications for tooth extraction were recorded. The diagnosis of split teeth was made from direct observation of the occlusal table or by applying a periodontal probe to exert force where there is an occlusal mesiodistal crack across both marginal ridges splitting the unrestored tooth in two segments. The extracted tooth was further examined to confirm those that were split teeth. Over the period of the study, diagnosis and tooth extraction were done by three dentists of more five years practice experience. The exclusion criterion for this study were split teeth with restoration. This is because longitudinal fractures have been observed to be more common in root canal-treated teeth, because the strength of the root canal-treated tooth has already been compromised by caries, restorations, or over extended access preparation [11], making it vulnerable to fracture. Further information on the likely cause of the fracture and how long it has been before seeking dental attention were elicited from patients with split tooth. Data was entered into Microsoft excel, sorted and exported into SPSS (SPSS version 16.0, Chicago, IL, USA) for analysis.

## Results

A total of 10192 patients comprising 6091 (60%) males and 4101 (40%) females were seen during the period of the study. A total of 762 teeth were extracted from 669 patients. Of which 594 patients had one tooth extraction, 65 patients-two teeth extraction, 6 patients-three teeth extraction, 1 patient-four teeth extraction, 2 patients −5 teeth extraction and 1 patient 6 teeth extraction. Extraction was most common in the third decade of life and also more common in females than males (Table 1). Dental caries and its sequelae was the leading reason for the extraction. Split teeth constituted for 39 (5%) of extracted teeth (Table 2). The 39 split teeth were extracted from 38 patients as one patient had two split teeth extracted on separate occasions from same patient. The majority 23 (61%) of extracted split tooth were done in patients in the fifth decade of life. More of the split tooth extraction were performed in males 28 (72%) than females 11 (28%) (Table 3).

**Table 1:** **Age and gender distribution of patients that had tooth extraction**

	Male	Female	Total
Age (years)			
1-10	32	42	74
11–20	25	54	79
21–30	86	95	181
31–40	59	56	115
41–50	58	51	109
51–60	61	38	99
61–70	9	3	12
Total	330	339	669

**Table 2:** **Reasons for tooth extraction among the patients**

Reasons	Frequency (n)	Percent (%)
Dental caries & sequelae	517	68
Recurrent pericoronitis in impacted 3^rd^ molars	64	8
Retained teeth	59	8
Split teeth	39	5
Fractured cusps	33	4
Periodontal disease	29	4
Supernumerary teeth	14	2
Trauma	7	1
Total	762	100

**Table 3:** **Age and gender distribution of patients with split tooth**

	Male	Female	Total
Age (years)		1	1
31-40			
41–50	17	6	23
51–60	8	4	12
61–70	2	**-**	2
Total	27	11	38

Overall mandibular teeth were more affected than the maxillary teeth and the most affected teeth were mandibular second molars 23 (59%) while the least affected were the mandibular first premolars 1 (3%) and the third molars 1 (3%) (Table 4). The reported masticatory accident as aetiology, were biting on stone 21 (53%) or piece of bone 10 (26%) while eating. A few 3 (8%) were suspected bruxists (Table 5). Most 25 (65%) of the patients gave a three to six month history of a traumatic accident and intermittent pain before presenting to the dental clinic for care (Table 6).

**Table 4:** **Pattern of split tooth in the patients**

Type of tooth	Frequency (n)	Percent (%)
mandibular second molars	23	59.0
mandibular first molars	4	10.0
maxillary second molars	3	8.0
maxillary second premolars	3	8.0
maxillary first molars	2	5.0
maxillary first premolars	2	5.0
mandibular first premolar	1	3
mandibular third molar	1	3
	39	100

**Table 5:** **Aetiology of split tooth in the patients**

Cause of split tooth	n (%)
Biting on piece of stone while eating	21(53)
Biting on piece of bone	10(26)
Cannot remember	5(13)
Suspected bruxist	3(8)
Total	39 (100.0)

**Table 6:** **Duration of symptom before dental visit in the patients**

Duration of symptom before dental visit	n (%)
1-6 days	3(8)
1–3 weeks	3(8)
1–2 months	8(20)
3–4 months	11(28)
5–6 months	14(36)
Total	39(100.0)

## Discussion

In this study, the prevalence of tooth extraction increased from the first decade of life climaxing in the third decade of life before declining thereafter in higher decades of life and most extractions were done on females than males. The reported reasons for teeth extractions in this study followed the pattern in previous studies in Nigeria and other parts of the world [12–15]. Dental caries and sequelae were the leading reason for tooth extraction. The increasing prevalence of dental caries due to the dietary and lifestyle changes with non-commensurate oral health preventive measures may be the explanation. Cumulatively, traumatic reasons in form of split tooth (5%), fractured cusps (4%) and other forms of trauma (1%) constituted 10% of the extracted teeth in this study highlighting traumatic event especially of cracked tooth variety as extraction prone event in the studied urban dental clinic. Periodontal disease was ranked as the fifth most common reason for teeth extraction which may be explained by the location of the study as periodontal disease is less common in urban than rural dwellers [16].

In this study, split teeth accounted for 5% of the total teeth extracted in the clinic. This means that one out of twenty teeth extracted was due to split tooth. This prevalence is considered reasonable despite the non-inclusion of split restored teeth. This reflects the increasing prevalence of cracks in intact unrestored teeth [17]. It is therefore important to improve early diagnosis of a cracked tooth to facilitate early reinforcement by castings with cusp coverage or by internal splinting with adhesive ceramic restorations in order to prevent the progression of the crack to a split [18]. Unfortunately, split tooth is an unstudied reason for tooth extraction thereby making comparisons difficult.

Overall, the majority of the split tooth was reported in the fifth decade of life. The loss of dentine elasticity and increased stress fatigue over time may have facilitated the increased occurrence of cracks in older patients [19, 20]. Unrestored split teeth considered in this study were found to be more common in males than females. In males, it was most common in fifth decade of life while in females, it was most common in the sixth decade of life. This can be explained by the higher occlusal forces recorded in men than women [21] because their masticatory muscles are more advanced in development [22].

Many morphologic and physical factors, such as deep grooves and pronounced intraoral temperature fluctuation may predispose posterior teeth to an incomplete fracture and subsequently to split tooth [18]. The prevalence of split tooth in this study was highest in the mandibular second molars (59%). The associated proximity to the temporomandibular joint based on the principle of the “lever” effect where mechanical force on an object is increased at closer distance to the fulcrum may be accountable for this [23]. Overall, the prevalence of split tooth in this study was higher in the mandibular molars when compared to the maxillary molars.

The pattern of crack and split tooth depend on the occlusion which have racial variations [17, 24]. It has been suggested that the prominent mesiopalatal cusp of maxillary molars could act as a plunger that induces structural fatigue in their mandibular antagonists [24]. The transverse ridge of the maxillary molars may provide structural reinforcement and account for the lower incidence of fractures in these teeth [25]. The maxillary molars and the maxillary premolars recorded a similar prevalence of 12.8% each while the mandibular premolar was 2.6%.

Importantly in this study, two split teeth were extracted from the same patient. It has been observed that patients who have an existing cracked tooth are likely to have other cracked teeth [26].

**Figure 1:**
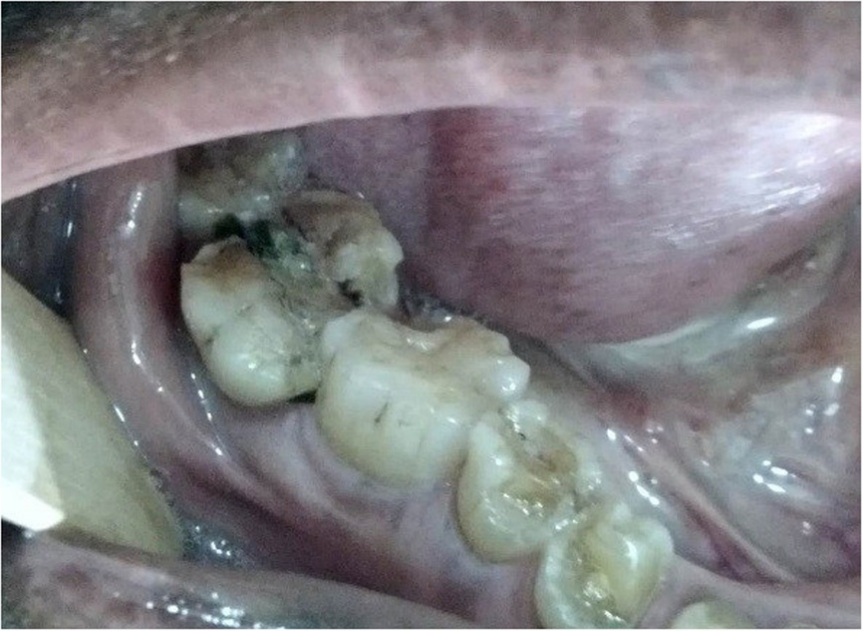
**Split tooth with food particles.**

**Figure 2:**
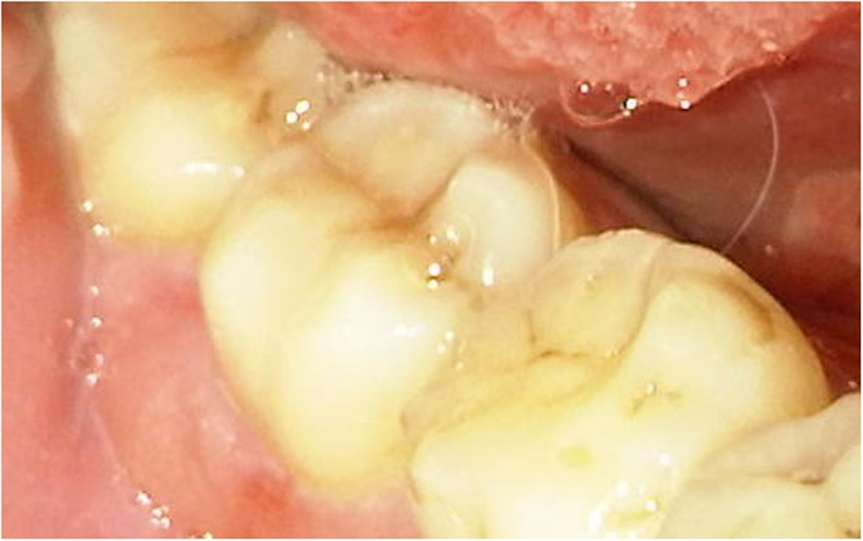
**Split tooth with periodontal abscess.**

**Figure 3:**
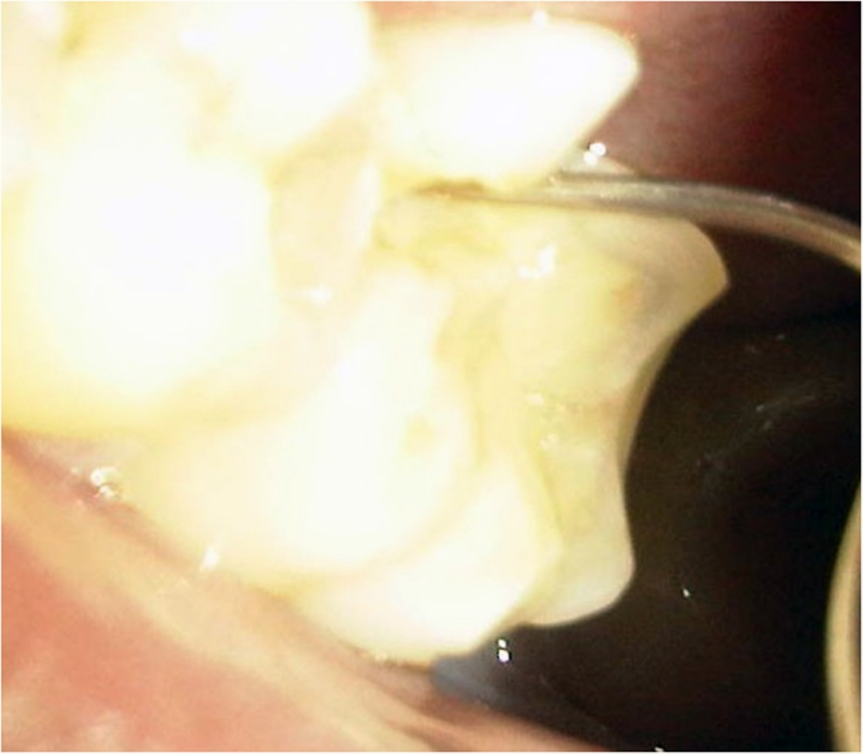
**Fractured cusp.**

**Figure 4:**
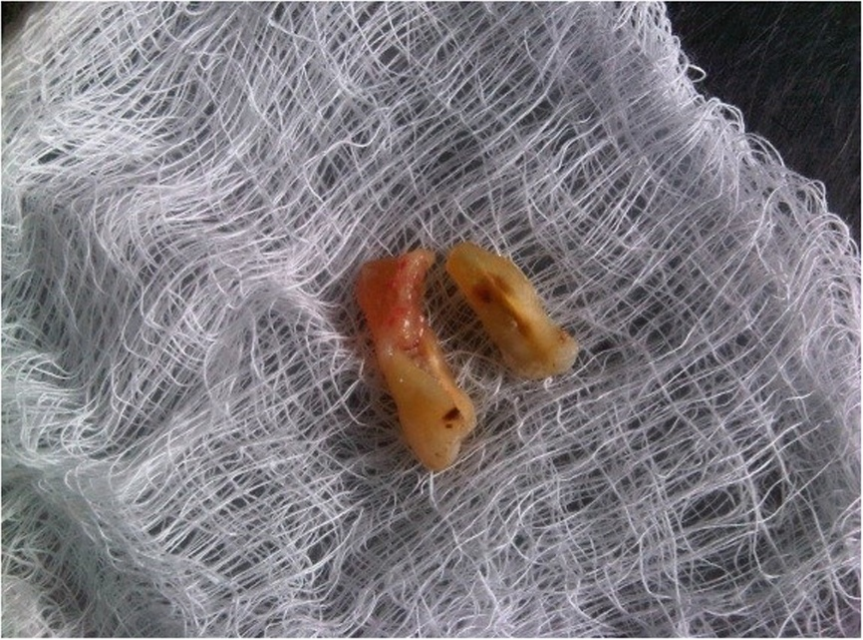
**Split tooth segments post extraction.**

In this study, patients gave a history of a masticatory accident and could remember biting hard on a piece of stone while eating a meal of rice, bread or beans. Others bit on piece of bone. A few of the patients were suspected bruxists. People of all ages are living more stressful lives, especially in urban centers which can result in crack-inducing habits like bruxism [26]. Unintentional biting with physiologic masticatory force on a small and very hard object may suddenly generate an excessive force due to the very small contact area that may cause the teeth to split [27]. Udoye & Jafarzadeh [20] observed that 10% of patients with cracked tooth syndrome had a history of masticatory accident as many factors related to cracked teeth are endemic to split teeth. Immediate pain during the incident which later subsided were reported and patients complained of marked pain on chewing and significant soreness of the jaw and gingiva. An important finding in this study was that most of the patients gave a three to six month history of a traumatic accident and intermittent pain. It implies that affected patients have either poor accessibility or high pain threshold which needs further investigation. When the patients finally presented in the clinic, the fracture line may be observed to be filled with food particles and exuding fluid (Figure 1), or to be separated but clean. Still others may present with the complication of an abscess (Figure 2). Figures 3 and 4 showed fractured cusp and split tooth post extraction respectively.

## Conclusion

Split tooth constitute a reasonable common reason for tooth extraction and this was most common in the fifth decade of life. High index of suspicion may improve early diagnosis of a cracked tooth in order to provide the correct treatment and patient management that will help relieve pain, restore function and improve the prognosis for the tooth and thereby, prevent the progression of the crack to a split.

## Authors’ information

IPO holds Bachelor of Dental Surgery (BDS) Degree and Fellowship of West African College of Surgeons (FWACS). She has been practicing as Specialist Oral and Maxillofacial Surgeon for more than a decade and currently practices as a Consultant Oral and Maxillofacial Surgeon at the Central Hospital Benin City, Nigeria.

CCA holds Bachelor of Dental Surgery (BDS) Degree, Master of Physiology (MSc Physio.) degree, Master of Public Health (MPH) degree and Fellowship of National Postgraduate College of Nigeria (FMCDS) lectures Periodontology in University of Benin and practices as a Consultant Periodontology at the University of Benin Teaching Hospital Benin City, Nigeria.
